# Supplementary material for: High power rechargeable magnesium/iodine battery chemistry
Source: Nat Commun. 2017 Jan 10;8:14083. doi: 10.1038/ncomms14083 (PMC5234091; doi:10.1038/ncomms14083)
Supplement: Supplementary Information — Supplementary figures, supplementary table and supplementary references. [file ncomms14083-s1.pdf]

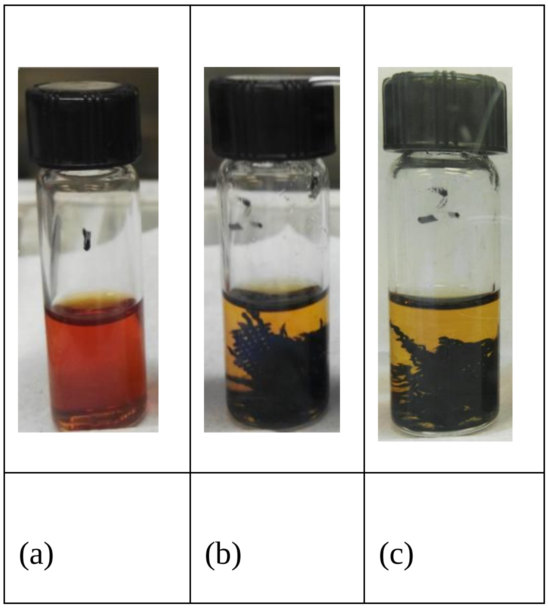

(a) (b) (c)

Supplementary Figure 1. The images of iodine solution in tetraglyme with/without active carbon cloth (ACC). (a) iodine solution; (b) iodine solution after adding ACC for 2 minutes; (c) iodine solution after adding ACC for 12 hours. ACC shows strong adsorption effect toward iodine. The color of the solution fades rapidly within 2 minutes after the addition of ACC, and does not change very much for the next 12 hours. The amount of iodine in the solution is equivalent to 1 mg iodine per cm^2 of ACC. The concentration of iodine in the solution is thus 39.3 mM.

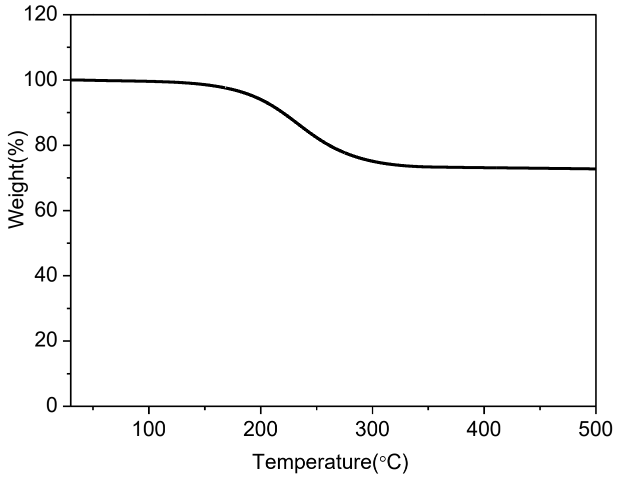

Supplementary Figure 2. Thermogravimetric analysis (TGA) of the ACC/I_2 cathode. This figures shows the result of a typical electrode with an iodine loading of 2.8 mg/cm^2.

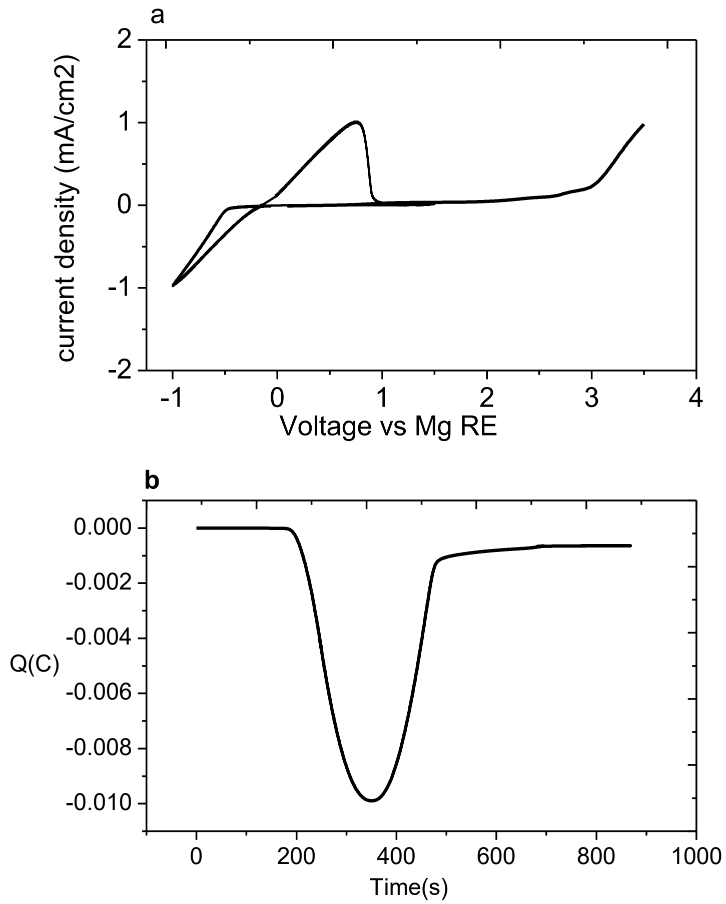

Supplementary Figure 3. Electrochemical performance of the electrolyte (2M Mg-HMDS in tetraglyme) in a three-electrode set-up. a) Cyclic Voltammetry (CV). Scan rate: 100 mV/s, voltage:-1V-3.5V. Working electrode: Pt, Reference electrode: Mg foil, Counter electrode: Mg foil. b) capacity-time curve. The basic properties of the electrolyte have been well documented in literature by Karger et al. [10] Here we conducted CV test in a three-electrode set-up to illustrate the Mg deposition/dissolution process and its electrochemical stability window. During the cathodic scan, Mg deposition starts at -0.5 V and deposition current increases rapidly with increasing overpotential. During the reverse scan, Mg stripping starts at -0.16 V. The Coulombic Efficiency of the deposition/stripping process can be evaluated by integrating current with time (Supplementary Figure 3b). The Columbic efficiency of 2M Mg-HMDS electrolyte is 94.5%. The electrolyte is quite stable until the voltage reaches > 2.7 V when oxidative current can be observed. Strong decomposition occurs when voltage exceeds 3.0V.

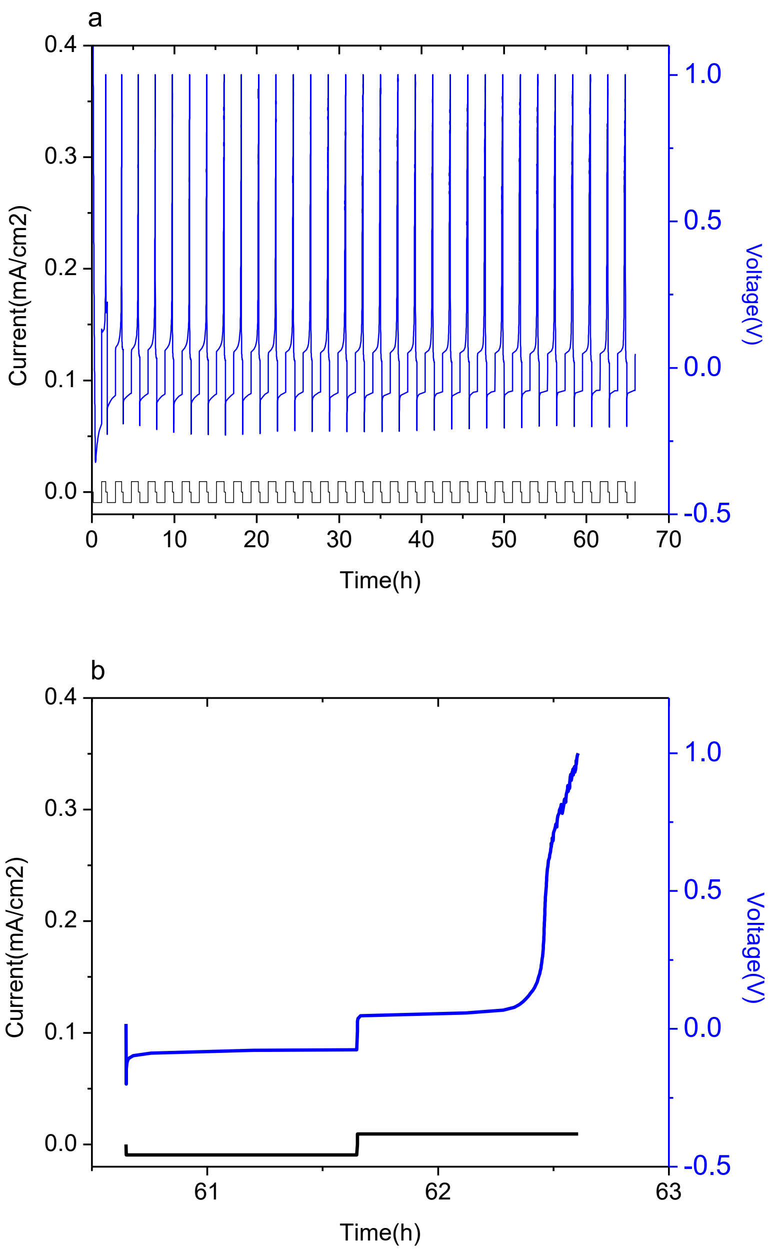
a
Current(mA/cm2)
Voltage(V)
Time(h)
0.4
0.3
0.2
0.1
0.0
1.0
0.5
0.0
-0.5
0
10
20
30
40
50
60
70
b
Current(mA/cm2)
Voltage(V)
Time(h)
0.4
0.3
0.2
0.1
0.0
1.0
0.5
0.0
-0.5
61
62
63

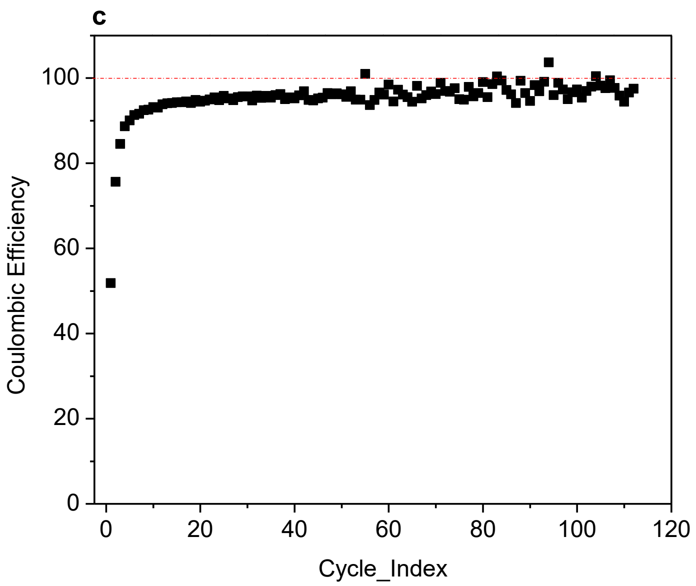

Supplementary Figure 4. Electrochemical performance of the electrolyte (2M Mg-HMDS in tetraglyme) in Cu|Mg coin cell. a) Galvanostatic discharge/charge. Current: 0.1 mA/cm^2. Discharge time: 1h. Charge cut-off voltage: 1V; b) A typical discharge/charge curve; During deposition, after the initial large overpotential to initiate the nucleation process, voltage stabilize at an overpotential below 0.1 V. During striping, overpotential is also below 0.1 V until the end of the charge. c) Coulombic Efficiency for Mg depositing/striping. After the initial activation cycles, an efficiency of close to 100% can be achieved.

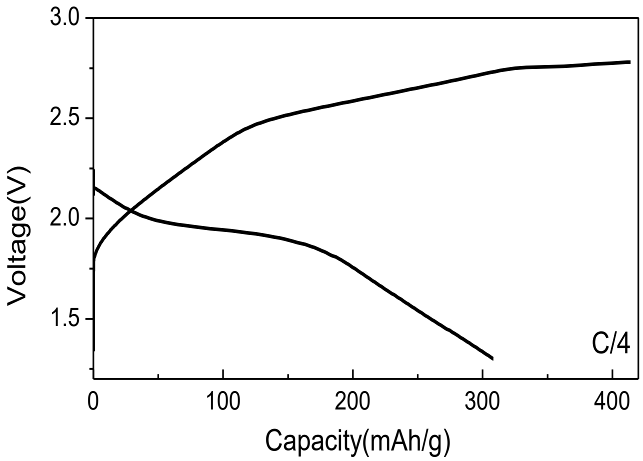

Supplementary Figure 5. Discharge/charge curve of ACC/I_2 cathode in 0.5M Mg-HMDS electrolyte. Current: C/4.

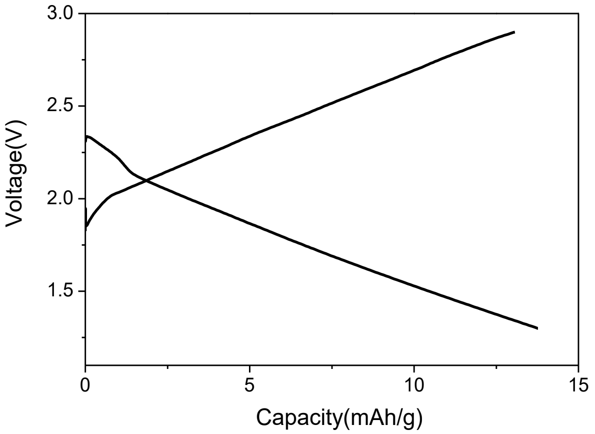

Supplementary Figure 6. Discharge/charge curves of the blank ACC. Voltage: 1.3-2.9 V, current: 52.7 uA/cm^2. The capacity is calculated based on the mass of ACC. This sloping profile suggests a capacitive storage mechanism, which corresponds to adsorption (during discharge) and desorption (during charge) of cations to the carbon/electrolyte interface. Because in a typical ACC/I_2 cathode the mass of blank ACC is ~7.8 mg/cm^2 and iodine mass is ~1 mg/cm^2. This capacitive storage of 14 mAh/g-carbon could be misinterpreted as an equivalent capacity of ~109 mAh/g-iodine for ACC/I_2 cathode discharged to 1.3V (Supplementary Figure 5), and ~62 mAh/g for ACC/I_2 cathode discharged to 1.6V (Figure 3a). For this reason, we intentionally subtract the contribution of capacitive storage from ACC when evaluating the iodine utilization in the composite electrode.

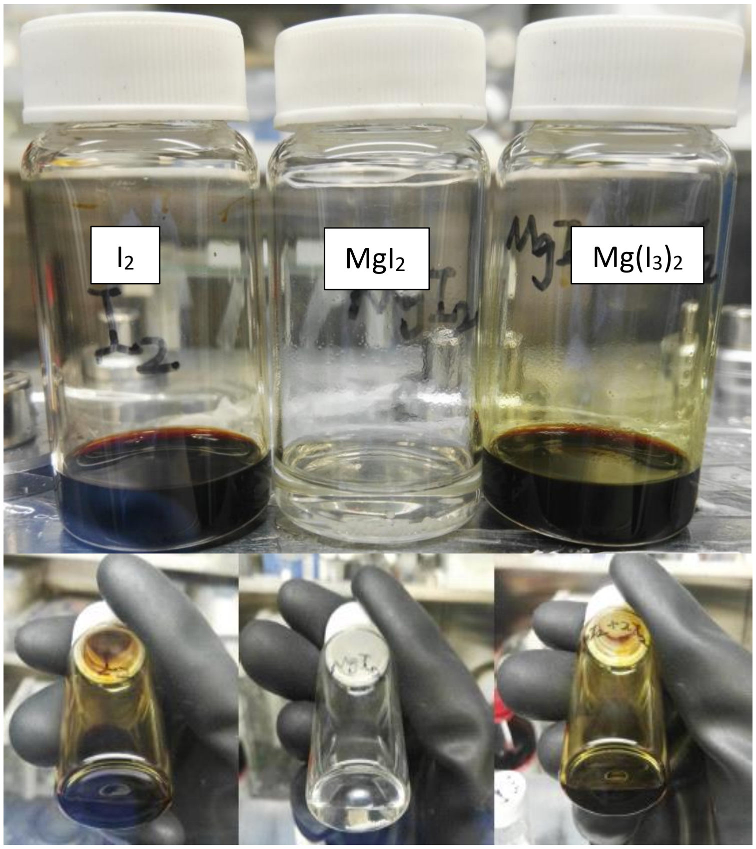

Supplementary Figure 7. Images of saturated I_2, MgI_2 and $Mg(I_3)_2$ solutions. Saturated I_2 and MgI_2 solutions are made by adding sufficient I_2 and MgI_2 into tetraglyme until precipitates show. I_3^-, the most common polyiodide species, was made by adding a mixture of I_2/MgI_2 ($I_2:MgI_2=2:1$) into the solvent.

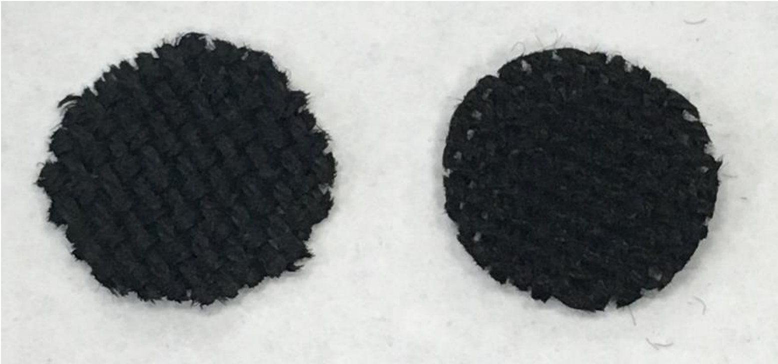

Supplementary Figure 8. Images of ACC/iodine cathode. left: after cycle; right: pristine. We can see through the pristine one since there are voids between the woven fibers (Figure 2b). However, in the cycled one the voids are all occupied by residual electrolyte. This morphology change of carbon is a result of long term immersing and hanging in solution instead of iodine reaction due to the weak connection between individual carbon fibers (Figure 2b). It is common for this kind of woven carbon cloth to lose their mechanical integrity when it is being soaked into a wettable liquid for long time because surface tension between individual fiber and the solution and gravity can be larger than the weak mechanical force between fibers. Actually, for all carbon/iodine cathode tested in Swagelok cell, where the electrode is being backed by a rigid current collector instead of being hanged in the electrolyte, no significant morphology change is observed (Supplementary Figure 8).

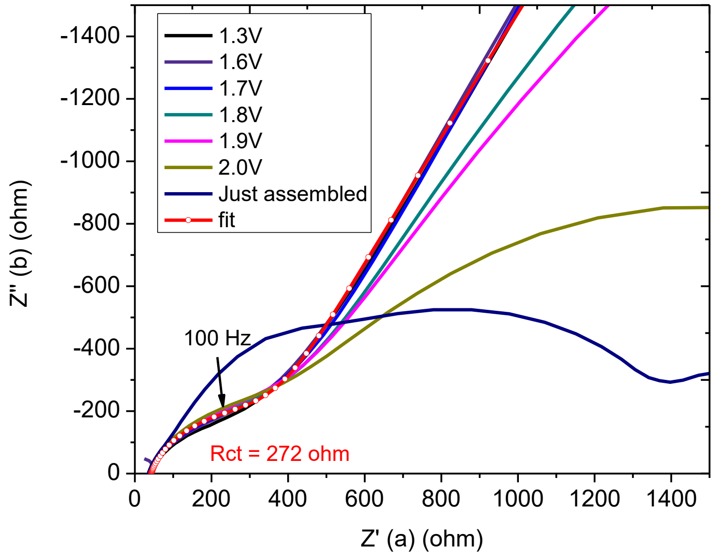

Supplementary Figure 9. Electrochemical Impedance Spectra (EIS) of the Mg/I_2 battery during discharge at different voltages. The battery is discharged to a certain voltage by CCCV (constant current then constant voltage) method before impedance measurement. The just assembled cell shows a large charge transfer resistance of > 1000 ohm, probably due to the activation energy required for initiating iodine → polyiodide phase transition. However, after the initiation, charge transfer resistance drops down by one order of magnitude to several hundreds. To get an accurate estimate of the charge transfer resistance, we fitted data at 1.7V and the charge transfer resistance for the iodine redox reaction is 272 ohm.

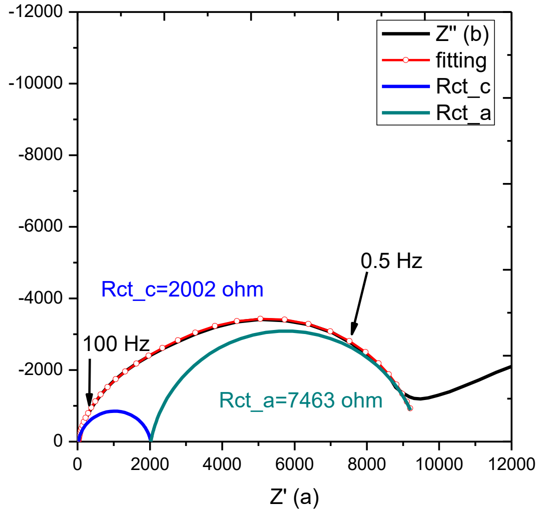

Supplementary Figure 10. EIS of Mo_6S_8/Mg battery

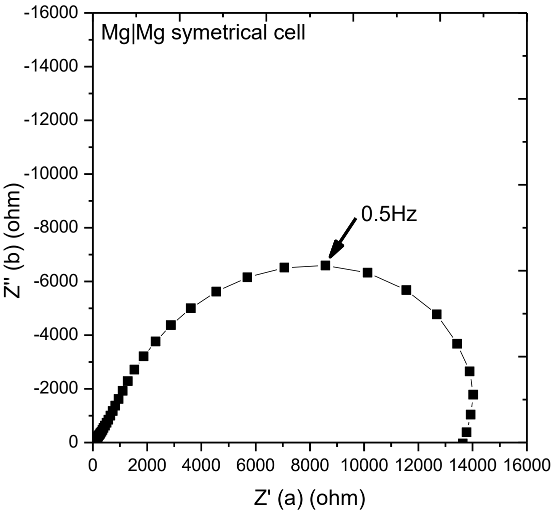

Supplementary Figure 11. EIS of Mg/Mg symmetrical cell. Impedance tests with intercalation material Mo_6S_8 as cathode were performed (Supplementary Figure 10), to compare the kinetics between a rocking-chair Mg battery and the Mg/I_2 system. The high and medium frequency response (10^6-0.1 Hz) was fitted into two separate semi-circles to represent the charge transfer resistance of the cathode redox reaction and anode redox reaction. The Mg/Mg symmetrical cell was built to identify the characterizing frequency for Mg deposition/striping in this electrolyte (Supplementary Figure 11), and it was found that the characterizing frequency for Mg anode redox reaction is 0.5 Hz. According to this, the medium frequency semi-circle(0.5 Hz) in Supplementary Figure 10 is assigned to Mg anode reaction in the Mo_6S_8/Mg battery, and the high frequency semi-circle(100 Hz) is assigned to cathode redox reaction. From the fitting result, the cathode charge transfer resistance for the rocking chair system is 2002 ohm, which is about one order magnitude higher than that of the Mg/I_2 battery. This further confirms the fast reaction kinetics of our Mg/I_2 battery.

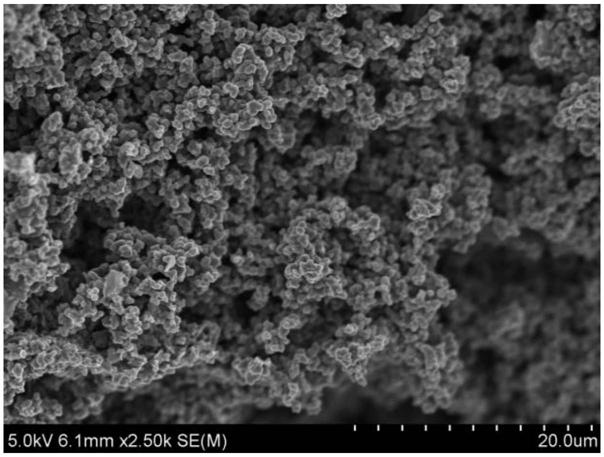

Supplementary Figure 12. Scanning electron microscopy (SEM) images of the MPC/I_2 cathode.

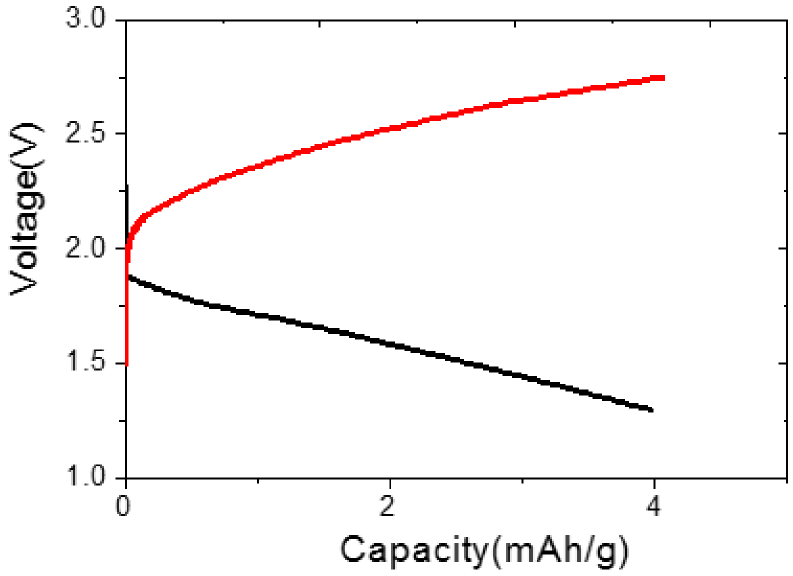

Supplementary Figure 13. Discharge/charge curve of blank (or neat) MPC in 0.5 M Mg-HMDS electrolyte. Current density: 52.7 uA/cm^2.Voltage:1.3-2.8V. In this work, the MPC/I_2 cathode has a typical iodine loading of 1 mg/cm^2, while MPC weighs 3 mg/cm^2. Just like other carbon material, MPC itself may provide some capacity by surface adsorption of ions. We intentionally conducted galvanostatic experiment with blank MPC cathode. The discharge/charge curve was given in Supplementary Figure 13. A typical electrochemical double layer capacitor behavior can be identified with a capacity of 4 mAh/g-carbon, which is equivalent to 12 mAh/g-iodine if this capacitive storage is misinterpreted as part of iodine's redox capacity. Nevertheless, in the discharge curve MPC/I_2, a total capacity of ~95 mAh/g-iodine can be obtained, suggesting that the contribution of carbon only takes a small portion. Indeed, a discharge plateau at ~2.1 V can be clearly identified, which is a strong sign of iodine reduction instead of carbon surface adsorption.

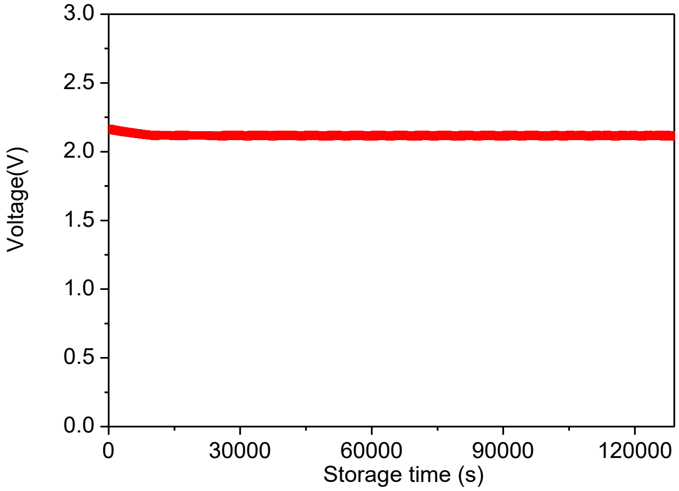

Supplementary Figure 14. Self-discharge test on Mg/I_2 cell with MPC/I_2 cathode. The OCV of the cells was monitored to evaluate the shuttle effect of the cell**.**

Supplementary Table 1 The capacity and voltage of typical RMB cathode materials.

Material Type	Material	Average Potential vs. Mg RE(V)	Capacity (mAh/g)	Energy Density (Wh/kg)	80% capacity retention(cycles)	reference
Sulfides	Mo_6X_8, X=S or Se	1.2	110	132	>100	[1]
	MoS_2	1.9	170	323	>50	[2]
	TiS_3	0.8	140	112	1	[3]
Oxides	V_2O_5	0.8	160	128	>50	[4]
	α-MnO_2	1.5	280	420	1	[5]
Polyanion	$MgFeSiO_4$	1.5	165	247.5	-	[6]
	$MgCoSiO_4$	1.6	260	416	> 30	[7]
Others	Fullerenes, C_{60}	1.5	50	75	1	[8]
	AgCl	1.8	176	316.8	2	[9]
Halogen	I_2	2.0	200	400	>120	This work

Supplementary References

1. Aurbach, D. *et al.* Progress in rechargeable magnesium battery technology. *Adv. Mater.* **19,** 4260–4267 (2007).

2. Liang, Y. *et al.* Rechargeable Mg batteries with graphene-like MoS2 cathode and ultrasmall Mg nanoparticle anode. *Adv. Mater.* **23,** 640–643 (2011).

3. Taniguchi, K., Gu, Y., Katsura, Y., Yoshino, T. & Takagi, H. Rechargeable Mg-ion battery cathode TiS3 with d–p orbital hybridized electronic structures. *Applied Physics Express* **9**, 011801(2016).

4. Tepavcevic, S. *et al*, Nanostructured Layered Cathode for Rechargeable Mg-Ion Batteries. *ACS nano* **9,** 8194-8205 (2015).

5. Zhang, R. *et al.* α-MnO 2 as a cathode material for rechargeable Mg batteries. *Electrochem. commun.* **23,** 110–113 (2012).

6. Orikasa, Y. *et al.* High energy density rechargeable magnesium battery using earth-abundant and non-toxic elements. *Sci. Rep.* **4,** 5622 (2014).

7. NuLi, Y., Zheng, Y., Wang, Y., Yang, J. & Wang, J. Electrochemical intercalation of Mg2+ in 3D hierarchically porous magnesium cobalt silicate and its application as an advanced cathode material in rechargeable magnesium batteries. *J. Mater. Chem.* **21,** 12437 (2011).

8. Zhang, R., Mizuno, F. & Ling, C. Fullerenes: non-transition metal clusters as rechargeable magnesium battery cathodes. *Chem. Commun.* **51,** 1108–1111 (2015).

9. Zhang, R., Ling, C. & Mizuno, F. A conceptual Mg battery with ultrahigh rate capability. *Chem. Commun.* **51**,1487-1490(2015)

10. Zhao-Karger, Z., Zhao, X., Fuhr, O. & Fichtner, M. Bisamide based non-nucleophilic electrolytes for rechargeable magnesium batteries. *RSC Adv.* **3,** 16330 (2013).
